# Application of JC1 for non-toxic isolation of cells with MDR transporter activity by flow cytometry

**DOI:** 10.1371/journal.pone.0174905

**Published:** 2017-04-05

**Authors:** J. Mario Wolosin, Aldo Zamudio, Zheng Wang

**Affiliations:** 1 Department of Ophthalmology, the Icahn School of Medicine at Mount Sinai, New York, NY, United States of America; 2 Black Family Stem Cell Institute, Icahn School of Medicine at Mount Sinai, New York, NY, United States of America; Hungarian Academy of Sciences, HUNGARY

## Abstract

The DNA intercalating dye Hoechst 33342 or its close analog DCV are actively removed from cells by the multidrug resistance transporter ABCG2, a protein overexpressed in metastatic cells and somatic stem cells. In bivariate blue-red flow cytometry fluorescent plots active Hoechst or DCV efflux combined with a concentration dependent bathochromic shifts of these nuclear dyes leads to the segregation of the transporter-rich cells into a distinct cell cohort tilted towards the shorter wavelength axis of the plot, the cohort is generically known as the side population (SP). This feature has facilitated the surface marker-independent isolation of live stem cells. A drawback, though, is the known toxicity of Hoechst dyes. In this study we show that JC1, a bathochromic mitochondrial membrane potential-sensitive dye applied at proper concentration, can yield flow cytometry fluorescent emission bivariate plots containing a low JC1 accumulation (JC1^low^) cohort. Using a combination of multiple cell lines, ABC-transporter inhibitors and viral vector-driven insertion of the ABCG2 gene or ABCG2 and ABCB1 shRNAs we demonstrate that JC1^low^ can be generated by either of the two aforementioned multidrug resistance transporters. Complete wash out of mitochondrial bound JC1 required more than 24 h. In spite of this tight binding, the dye did not affect either the mitochondrial membrane potentials or the proliferation rate. In contrast, contemporaneous with its nuclear accumulation, Hoechst 33342 or DVC, caused changes in the fluorescent emission of mitochondrial membrane potential sensitive dyes resembling the effects caused by the mitochondrial uncoupler FCCP. In a number of cell lines exposure to Hoechst resulted in marked slow-down of proliferation and abolition of ABCG2 transport activity during the subsequent 2 days but in K562 cells the exposure induced cell extended death. Overall, its lack of toxicity vis. a vis. the toxicity and genotoxicity of the DNA intercalating dyes makes JC1 an ideal tool for isolating live cells expressing high multidrug resistance transport activity.

## Introduction

The incubation of live cells with the DNA binding supravital dye Hoechst 33342 (Hoechst) frequently results in flow cytometry bivariate 450/670 nm emission plots incorporating small cell cohorts displaying, a) lower absolute fluorescence, and b) a higher 450/670 nm ratio than the main cell population. This pattern results from an accumulation-dependent red emission (bathochromic) shift of the DNA-bound Hoechst, once its DNA intercalation reaches a certain threshold, allowing multimeric spectral interactions. The cohort with reduced stain results from active efflux of the dye by the ABCG2/BCRP transporter. Due to this higher 450/670 nm ratio, the Hoechst-exclusion cohort is referred to as a (blue) side population (SP). It was initially demonstrated that in bone marrow the SP represents a unique subset of hematopoietic stem cells [1]. Later it was shown that the ABCG2 dependent Hoechst exclusion is a feature of stem cells of many organs and tissues [2].These developments opened the door for the flow cytometry-based isolation of live stem cells in a variety of cellular systems, including ocular surface epithelial lineages [3–6]. Hoechst can be replaced by Due Cycle Violet (DCV), a structural analog allowing excitation by the 405 nm diode laser instead of the UV laser [7].

Previously, we have shown that incubation of ocular surface epithelial cells with the mitochondrial membrane potential (MMPT) sensitive dye JC1 [8, 9] results in bivariate 525/585 nm green/orange emission plots (JC1 image) containing a low dye accumulation cohort (JC1^low^) situated to the green-side of the main cell cohort [6,10] which thus constitute a JC1-SP. This cell subset was efficiently abolished by the ABCG2 specific inhibitors fumitremorgen C (FTC) and Ko143 [11, 12] and contained the great majority of clonogenic cells within the outgrowths that develop from limbal biopsies explants [10] Thus, we and others [13] have used this JC1 green-side population as an alternative means to identify and/or quantitate ABCG2 transport activity in the ocular surface epithelial cells.

The studies in the ocular surface epithelia have been facilitated by the remarkably high levels of ABCG2 expressed by the cells of the limbal outgrowth [14, 15]. However, efflux rate measurements in cells expressing exclusively ABCG2 or ABCB1 have shown that JC1 can serve as substratum for either multidrug resistance (MDR) transporter [16–18]. Thus, to determine the role of MRD transporters in the generation of JC1-SPs we have now performed an extensive study on the application of JC1 for the cytometric isolation of cells displaying MDR transporter activity and assessed the putative biological value of the JC1 approach in a study employing multiple cell lines of disparate origin. The study includes a comparison of biological effects of cell exposure to JC1, Hoechst 33342 or DCV. Our results indicate that, a) JC1 is transported with comparable efficiency by the two main MDR transporter proteins, ABCG2 /BCRP and ABCB1/p-glycoprotein; b) JC1 washout is remarkably slow with a half time of dissociation exceeding 24 h; c) in spite of this prolonged residence in the mitochondria the JC1 did not have any measurable late effect on either MMPT, gross physical cell parameters or cell proliferation of transformed and tissue derived cell lines; and d) unlike JC1, incubation with Hoechst 3342 or DCV at the concentrations and periods used for the identification of SPs markedly affected the MMPT during or after the dye incubation period and resulted in either delay in reduced marked proliferation rate proliferation in most cell line studied and cell death in K562 cells.

## Materials and methods

### Materials

JC1 was purchased from Axxora (San Diego, CA). Dulbecco’s Modified Minimal Essential Medium (DMEM), DMEM/Ham F12 (-F12) and DKSFM culture media and DCV were obtained from Invitrogen. Fetal bovine serum (FBS) was procured from Atlanta Biologicals (Atlanta, GA). All other reagents were purchased from Sigma-Aldrich (St. Louis, MO).

### Cell culture

A SV40-adenovirus-immortalized human corneal epithelial cell line (HCEC or HCE cells) was generously gifted by Dr. Araki-Sasaki, (Kagoshima Miyata Eye Clinic, and Kagoshima, Japan). The widely available Caco-2, HEK293T (293T) and K562 cell lines were obtained in house. HCE cells were cultured in 90% DMEM-F12-10% FBS. Caco-2 and 293T cells were cultured in DMEM-10% FBS. The blood-derived K562 cells were cultured in suspension in 90% RPMI-10% FBS. Primary culture cells were generated from freshly collected abattoir tissue. Limbal epithelial (pLiE) cells were generated by culturing rabbit limbal superficial biopsies on 4 μm-pore, 6-well Transwell inserts in human supplemented epithelial medium (SHEM; contains per liter, 945 mL of a 1:1 mix of Dulbecco’s Modified Minimal Essential Medium and Ham-F12 (DMEM-F12); 50 mL fetal bovine serum (FBS), 1X insulin-transferrin-selenium mix; 10 μg cholera toxin; 5 μg epidermal growth factor and; 28 mg phosphoethanolamine). After 10–12 days, the biopsies yield 2 cm-diameter pure epithelial outgrowths containing 2–5 x 10^5^ cells displaying a very high percentile of JC1-SP cells (10). Outgrowth cell sheets were separated from membrane inserts by incubation with 2 mg/ml Dispase II (Roche, Nutley, NJ) made in phenol-red and bicarbonate-free, Hepes-buffered DMEM-F12 (HDMEM-F12) for 30–40 min under moderate orbital rotational motion at 37°C. Single cell suspensions were then generated by a short incubation in trypsin and resuspension in DMEM-F12 with 5% FBS. Fresh rabbit conjunctival epithelial cells were prepared by sequential Dispase-Trypsin digestion as described [19], resuspended in DMEM-F12 with 5% FBS and used immediately. Primary cultured keratinocytes (pcKs) were generated from rabbit inner ear by the same sequential Dispase-trypsin protocol used for conjunctiva and cultured and treated with reagents in DKSFM medium.

### Generation of cell lines expressing of shRNAs or ABCG2

Lentiviral vector for stable expression of shRNA sequences were generated using the Sigma Mission^R^ plasmids for stable inhibition of ABCB1 or ABCG2. For the ABCB1 we used clones TRCN0000059684 (ABCB1-A; brings down ABCB1 mRNA expression in lung A549 cells by 95%, as per manufacturer literature) and TRCN0000059683 (ABCB1-B). For ABCG2 the clones were TRCN0000059802 (ABCG2-A) and TRCN0000059801 (ABCG2-B). A lentiviral plasmid for the expression of ABCG2 (pSIN4-EF2-ABCG2-IRES-Neo) was obtained from Addgene. Viral particles were generated by lipofection (Lipo293, SignaGen, Rockville, MD) of 293T cells with a mix of 1 μg ABCG2 plasmid and 3 μg of packing plasmids (UMIX™; Biogenova, Potomac, MD). Replication-deficient viral particles were collected by centrifugation of supernatant at 25,000x g for 5 h, resuspended in DMEM and used to transduce cells in FBS-free medium with the help of 8 μg/ml polybrene. Transduced cells were selected by incubation of cultures with puromycin (shRNAs) or genticin (ABCG2), under conditions that resulted in complete elimination of un-transduced cells.

### Dye loading and flow cytometry

For the generation of bivariate images, unless indicated otherwise, adherent cultures at 20–30% density or less than 200,000 cell per ml for the K562 cells were complemented with 250 nm JC1 or 4μg/ml Hoechst or 5 μM DCV. MDR inhibitors were introduced 60 min before the addition of any one of the three transport reporting dyes to ensure intracellular equilibration. At the end of the dye incubation period, adherent cells were trypsinized for 5–7 min. Trypsinization was stopped with an equal volume of 90% HDMEM-F12- 10% FBS-2 μg/ml propidium iodide (PI). K562 cells were diluted 10-fold with cold HDMEM-F12, spun down, and resuspended in 95% HDMEM-F12- 5% FBS-1 μg/ml propidium iodide. Analytical flow cytometry was performed in either an Accuri 6 or a LSR II cytometer (BD). Sorting was done in a FacsAria II instrument (BD). The fixed gain feature of the Accuri 6 makes this cytometer ideal for comparisons of results from day to day, as there is no risk of inadvertent gain changes or drifts. JC1 emissions from excitation at the 488 nm were collected at 525 nm (Fl1; JC1 green) and 585 nm (Fl2, JC1 orange) and at 620 nm (Fl3) for PI. Customarily, studies where JC1 is used to track cellular MMPT set the shorter emission wavelength of the bivariate plot in the X axis. In the present study we set the shorter wavelength in the Y axis to establish bivariate images resembling those observed with Hoechst. The equivalent emission wavelengths for Hoechst or DCV were 450 (blue) and 670 (red) nm, respectively. Gates, including the final gate for dye excluding cells were subjectively set based on the flow cytometry images, however, within each experiment the same gate settings were used to determine dye exclusion cohort percentiles changes that resulted from experimental maneuvers. Tests demonstrated that increasing or decreasing the stringency of the dye excluding gate selection which proportionally changed the % of cells within the gate, had very minimal effect on the percentile change induced by any given experimental manipulation within each experiment.

To determine putative effects of Hoechst on MMPT during its accumulation in cells, 10^6^ cells/ml K562 cells were incubated with either 5 nM 3,3'-Dihexyloxacarbocyanine Iodide (DiOC(6)-3) for 30 min or with 100 nM Rhodamine 123 (Rho123) for 60 min. Pilot test showed that these times ensure their full dye equilibration in mitochondria. DiOC (6)-3 reports MMPT decreases by respective decreases in fluorescent signal intensity due to dye accumulation loss [20]. Rho 123, as applied here, reports MMTP decreases as fluorescence increases due to high quenching and red spectral shifts for this dye in the high MMPT state [21].Cultures were then complemented with either 5 ug/ml Hoechst 33342 or 10 μM Carbonyl cyanide-4-trifluoromethoxy) phenylhydrazone (FCCP) and 1 μg/ml PI and changes in Fl1 signal in live cells were measured at selected times for up to 90 min. Accuri 6 results are presented as displayed by the instrument software. The LSR II FCS files were processed in FCSExpress^tm^ (De Novo Software, Glendale, CA). All results are presented as dot plots of small cell numbers (2,000 to 4,000) to allow for detailed visualization and are representative of between 2 and 4 independent repeats.

To determine the relative concentration of ABCB1 transporters with a cell surface antibody on live 293T cells, sparse cultures were washed thrice with phosphate buffered saline (PBS) and incubated in the last wash for 5–10 min to elicit cell detachment. Detached cells were complemented with one volume of trypsin, incubate at room temperature for 2 min followed by addition of 10% FBS. The cells were then spun down resuspended in 100 μl Dulbecco’s PBS-1% bovine serum albumin (BSA) and 1 μg/ml of either FITC-conjugated anti-ABCB1 monoclonal antibody (Clone UIC2, Abcam, Cambridge, MA) or FITC-conjugated isotype control (Abcam). After 45 min incubation at 4°C, cell suspensions were diluted 20-fold with PBS- 1% BSA and 15 min later fluorescence distribution was determined in the Accuri 6 instrument.

### Microscopy

Images of live cultures maintained at 37°C in HDMEM/F12 and incubated with dyes for various times were captured in an Olympus IMT-2 inverted microscope equipped for epifluorescent illumination with a 100 W mercury lamp and a dichroic filter for excitation at 490 ± 10 nm and wide 525–620 nm emission range. Under these conditions, the JC1 emission in normal cells appears as an orange fluorescence and after induced depolarization as green fluorescence. Hoechst and DCV fluorescence were visualized with a dichroic filter cube for DAPI. Images were captured with a Nikon D90 camera.

### Western blot

Lysates from HCE cells were prepared, and protein was electrophoresed and electro transferred to a PVDF membrane as described [21]. Membranes were blocked with 5% fat-free milk in 1x PBS buffer-0.1% Tween-20 for 1 h, incubated overnight at 4°C with a 1 μg/ml rabbit polyclonal antibody against ABCG2 (Abgent, San Diego, CA) and subsequently with a 1:2000 dilution of HRP-conjugated goat anti-rabbit IgG for 1 h. Bound HRP activity was visualized using ECL Plus (GE Healthcare, Piscataway, NJ). Chemiluminiscence was captured with X-ray film. The membrane was then stripped and processed in the same manner for the detection of GAPDH protein.

### Proliferation assays

Cultures at 20–30% density were incubated with 250 nM JC1, 5 μg/ml Hoechst 33342 or 5 μM DCV for 75 min under the same conditions used to dye load the cells for flow cytometry analysis. Following wash out of these effectors, cells were cultured for between 0 to 4 days and after harvesting they were resuspended in 90% HDMEM-F12- 5% FBS-1 μg/ml PI and live cells were counted in the Accuri 6 flow cytometer.

## Results

### JC1 accumulation and bivariate emission

**Fig 1:**
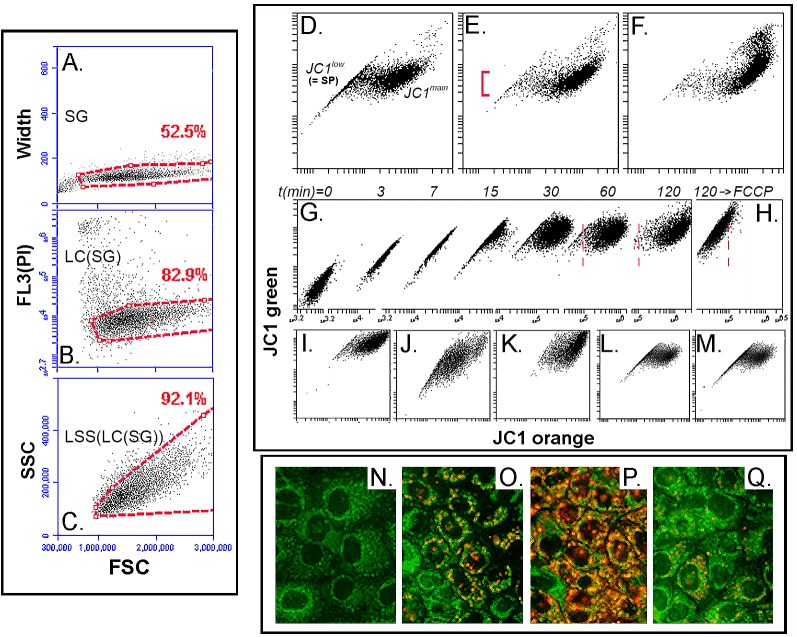
Bivariate JC1 flow cytometry plots and correlated micrographs of HCE cells as a function of dye concentration, incubation and wash out time, re-incubation and re-culture of presorted JC1-SP and JC1-nonSP populations. **A-C**. Graphical description of the gating strategy applied to all samples. Width vs. forward scatter (FSC) was used to exclude cells deviating from the main cohort of single cells (A), a plot of Fl3 (PI) fluorescence vs. FSC was used to select the live cells within the single cell population (B) and the final gating action excluded cells with high SSC/FCS ratios because those likely to be in early stages of apoptotic death (C). **D-F.** JC1 images after incubation with 125 (D), 250 (E) or 500 (F) nM JC1 for 60 min. The JC1^low^ and JC1^main^ cell cohorts are indicated in D. The vertical bracket in E marks the wide range of 525 nm emission intensities over which the transition from JC1^low^ to JC1^main^ occurs for different cells. **G.** Composite of bivariate images for HCE cells incubated in 250 nM JC1 as a function of time of incubation. **H.** JC1 image after incubation for 120 min with 250 nM JC1 including 10 μM FCCP for the last 20 min. Note the x-axis scales and vertical red dashed lines indicating the stain position after FCCP relative to the position of the JC1^low^ and JC1^main^ prior to FFCP exposure. **I.** JC1 stains 1 h after 60 min incubation with JC1. **J.** Same culture as in panel I after 18h incubation in JC1-free conditions. **K.** Same sample as J after re-incubation with JC1 for 45 min. **L** and **M.** JC1 images after one week culture of sorted JC1^low^ (L) and JC1main (M) cells. **N-Q.** Fluorescence images for the HCE cells incubated for 5 (N), 15(O) or 60 (P) min with 250 nM JC1 or as P but with the inclusion of FCCP for the last 15 min (Q).

Fig 1A–1C shows the gating strategy used in this study and the basic features of JC1 accumulation in HCE cells as reflected in green/orange bivariate plots. HCE cells were selected for these preliminary characterizations because they were known to us to exhibit only moderate levels of JC1 efflux activity which could be fully overcome by increasing dye concentration and/or incubation time. Thus, they are very useful to illustrate the relationship between extent of dye loading and bivariate plot appearance. Fig 1D–1F shows the effect of 125, 250 and 500 nM concentrations after 75 min incubation. Based on these results, we selected a 250 nM concentration for all subsequent studies to characterize JC1 exclusion behavior. Fig 1G is a composite depicting the changes in the bivariate plot image, as the dye accumulates in HCE cells with increasing incubation time when using 250 nM JC1. During the initial dye accumulation period, the 525/585 nm intensity increases at a constant ratio. However, within a certain range of JC1 accumulation, after about 15 min, a bathochromic transition starts developing. This JC1 red shift represents aggregation driven by the MMPT. The bathochromic shifts increase over time until at 60 min only a small set of less rapid dye accumulating cells maintains the initial green/orange ratio. Full melding of these cells with the main population occurred after 120 min, suggesting that in HCE cells the laggard or JC1-excluding cells represent cells with an efflux capacity that is only moderately higher than the general cell population. When the MMPT uncoupler FCCP [22] was included during the last 20 min of the 120 min incubation, the bathochromic shift characteristic of the JCP-SP cell set was nullified (Fig 1H); the 525/585 ratio reverted to the value observed during the first few minutes of incubation, albeit at higher absolute values. Overall, these results indicate that development of a side population pattern with the mitochondrial dye results from the fact that, while the MMPT drives the intra-mitochondrial JC1 aggregation associated with the bathochromic shift, it can elicit the latter effect only if the dye concentration reaches a certain minimal threshold. Cells that actively exclude JC1 may either not reach this threshold or reach it at a much later time in the incubation period. The transition of individual cells from the JC1^low^ to the JC1^main^ cohort always occurs over a relatively wide 525 nm emission intensity (Fig 1D). One factor that may contribute to this pattern are differences in average MMPT values between different cells, i.e., the higher the average MMPT the lower the concentration threshold at which JC1 undergoes aggregation.

For dye staining to be useful for flow cytometry applications, a sufficiently long retention time is necessary. Hence, we examined JC1 washout rates. Cells maintained in culture for 60 min at 37°C after JC1 removal from the medium prior to trypsin harvesting displayed bivariate plots that were essentially identical to the plots of cells harvested immediately after the end of 60 min of JC1 exposure (not shown), indicating a very tight JC1 attachment. Eighteen h after the incubation with JC1, the average green channel intensity decreased to about 40 ± 8% (n = 6) of the intensity measured 60 min after the JC1 incubation (compare Fig 1I and 1J). Re-incubation with 250 nM JC1 for 45 min at this point fully restored the fluorescence intensity and green/ red emission ratio (Fig 1K). These results demonstrate that, a) JC1 is well retained in the mitochondria, and b) in spite of the continuous presence of JC1 the MMTP is not affected to any visible level. When the JC1 loaded HCE cells were sorted into the JC1^low^ and JC1^high^ subpopulations and these subpopulations were re-cultured for one week, the JC1 bivariate plots for the previously sorted populations were essentially indistinguishable from one another (Fig 1L and 1M). This behavior suggests that in these immortalized cells the presence of the JC1^low^ cohort reflects transient fluctuations in the expression levels of efflux transporters, rather than the existence of bona fide distinct, stable subpopulations.

The microscopic examination of HCE cells at various points in the JC1 loading process is depicted in the last four panels of Fig 1. After 5 min incubation (Fig 1N) there was a fine green granularity throughout the cytosol on a diffuse green background. By 15 min, red granules became incipient in some cells (Fig 1O) and by 60 min (Fig 1P) orange-red puncta became a prominent feature. Consistent with the flow cytometry results, the addition of FCCP for the last 15 min of the JC1 incubation converted most of the orange puncta to green puncta (Fig 1Q).

### Effect of MDR transporter inhibition or gain of function on JC1 and Hoechst images

**Fig 2:**
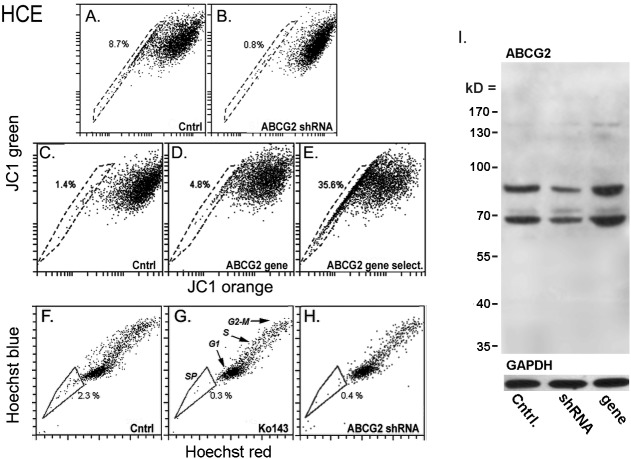
Effect of inhibitors or shRNAs for ABCB1or ABCG2 transporters on JC1 or Hoechst exclusion in HCE cells. All cells were incubated for 60 or 75 min with either 250 nM JC1 or 4 μg/ml Hoechst, unless indicated otherwise. Inhibitors were added 60 min before the addition of JC1 or Hoechst. The % of cells included within subjectively selected JC1-SP or Hoechst-SP domains (gates) are indicated next to the gate. **A** and **B.** Sixty min incubation with JC1; control (Cntrl) and puromycin-selected cells transduced with the ABCG2-A shRNA. **C-E.** Seventy five min incubation with JC1 incubated for 75 min; Control (C) and ABCG2 gene transduced cells before (D) or after (E) antibiotic selection. Note that the shRNA, in addition to the elimination of the JC1^low^ cohort, causes a rightward shift of JC1^main^ cells (B) while conversely, addition of the ABCG2 gene causes not only a large increase in the JC1^low^ but also and a leftward shift of JC1^main^ cells (E). **F-H.** Hoechst stain for 75 min. **F. C**ontrol cells. **G.** Cells pre-incubated with 2 μM Ko143. The G0/G1, S and G2-M cell cohorts are indicated. **H.** ABCG2 shRNA transduced and puromycin selected cells. **I.** Western blot of unmodified HCEC and HCEC transduced with ABCG2 shRNA (shRNA) or ABCG2 gene. Top: ABCG2 stain. Bottom: GAPDH stain of the same blot.

To identify transporters responsible for the establishment of JC1 exclusion cohorts we used a combination of gene expression techniques for gain or loss of function and transport inhibitors in four cell lines. Transduction of the HCE cells with an ABCG2 shRNA reduced the JC1-SP and shifted the main cohort rightward (Fig 2; compare A with B), indicating the involvement of ABCG2 in the dye exclusion activity. In contrast to the effect of the ABCG2 shRNA, the JC1-SP cohort was not affected by transduction of these cells with two distinct ABCB1 shRNAs or by Zosuquidar (2 μM), a specific inhibitor of ABCB1. The JC1-SP was also unaffected by MK571 (10–40 μM) a specific inhibitor of ABCC1/MRP1 (not shown). Finally, HCEC transduction with the ABCG2 gene resulted in a large increase in the size of the JC1-exclusion cohort and a leftward move of the main cohort (Fig 2; compare C with D and E).

Incubation of HCE cells with 4 μM Hoechst 33342 for 75 min generated an SP (Fig 2F). Pre-incubation with Ko143 (2 μM) reduced the number of cells within the arbitrarily defined Hoechst-SP domain by 83% ± 15 (n = 4) and transduction with ABCG2-A shRNA completely removed cells from the defined SP domain (Fig 2H and 2I). Finally panel I shows the actual changes in total ABCG2 polypeptide content following the transduction with an ABCG2 shRNA or gene and the GAPDH content in the same blots. The ABCG2 stain consisted of two similar intensity bands within each specimen. This pattern is consistent with the known effect of ABCG2 glycosylation on its on electrophoretic mobility [23]. The changes in protein amount caused by the shRNA or ABCG2 gene transductions, whether base on the top or bottom band, or both are qualitatively consistent with the changes in function.

**Fig 3:**
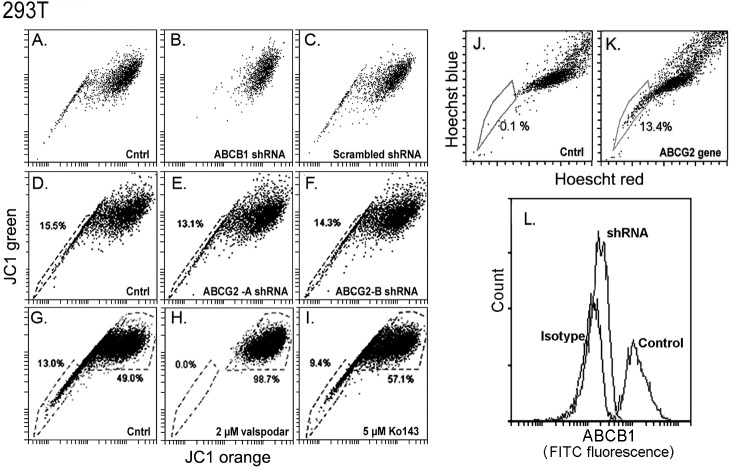
Effect of inhibitors or shRNAs for ABCB1or ABCG2 transporters on JC1 or Hoechst exclusion in 293T cells. Cells were incubated with dyes for 75 min. **A-C.** Control (A), and ABCB1shRNA transduced (B) or and scrambled shRNA transduced cells (C). **D-F**. Control cells and cells transduced with the ABCG2-A or ABCG2-B shRNAs, respectively. **G-I.** Control medium (G), medium complemented with 2 μM of either valspodar (H) or Ko143 (I), respectively. **J and K.** Hoechst 33342 stain in control (J) or after transduction with the ABCG2-A gene (K), respectively. **L.** Flow cytometry histogram of control or ABCB1shRNA-transduced live 293T immunostained with FITC-conjugated anti-ABCB1 antibody. An FITC conjugated isotype control was used to confirm the specificity of the staining.

The rest of the transformed cell lines and primary epithelia tested, including 293T, Caco-2 and K562 cells, displayed JC1 excluding activities that were clearly more robust than the activity in HCE cells. Fig 3A depicts JC1 bivariate emission images for 293T cells following 60 min incubation with JC1. Transduction with ABCB1-A shRNA resulted in the complete elimination of the JC1-SP (Fig 3B). In contrast, the degree of dye exclusion was unaffected by either a scrambled sequence shRNA (Fig 3C) or transduction with the two different ABCG2 shRNAs (Fig 3D–3F). Consistent with the shRNA effects in these cells, the ABCB1 inhibitor valspodar eliminated the JC1 exclusion cohort (Fig 3; compare G and H) whereas the ABCG2 inhibitor Ko143 had a minimal effect (Fig 3I). FTC (1μM) was similarly ineffective in these cells (not shown). These results demonstrate that in the 293T cells the generation of a JC1-SP occurs primarily or solely as a result of the activity of the ABCB1 transporter. The residual effect of Ko143 may in fact be due to a weak inhibitory effect of Ko143 on ABCB1 [24], a feature shared by FTC [25]. Consistent with this hypothesis, the 293T cells lacked a Hoechst–SP (Fig 3J), whereas the same cell type generated a large exclusion cohort when transduced with the ABCG2 gene (Fig 3K). Finally, we used a cell surface antibody against ABCB1 to confirm that the transduction of the 293 T cells with ABCB1-A shRNA essentially eliminated surface expression of this transporter (Fig 3L).

**Fig 4:**
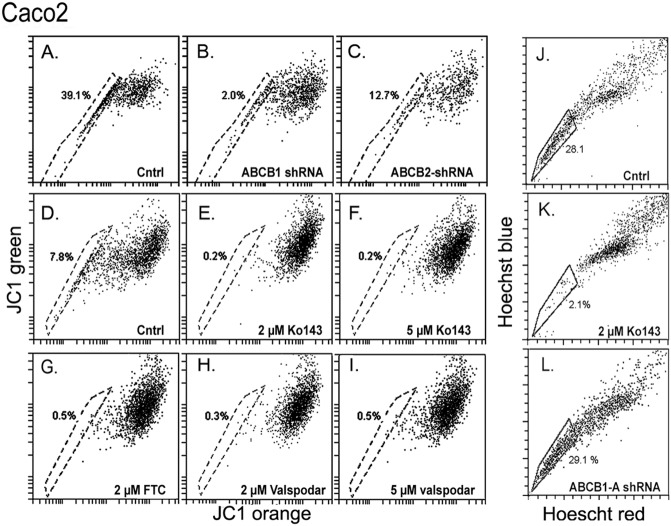
Effect of inhibitors or shRNAs for ABCB1or ABCG2 transporters on JC1 or Hoechst exclusion in Caco-2 cells. **A-C.** Control (A), ABCB1-A shRNA (B) and ABCG2-A shRNA transduced (C) cells were incubated with JC1 for 75 min. **D-I.** Control cells (D) and cells pretreated with 2 μM Ko143 (E), 5 μM Ko143 (F), 2 μM FTC (G), 2 μM Valspodar (H), or 5 μM valspodar (I) were incubated with JC1 for 90 min. **J-L.** Control cells (J), and cells pretreated with 2 μM Ko143 (K) or transduced with ABCB1-A shRNA (L) were incubated with Hoechst for 75 min.

**Fig 5:**
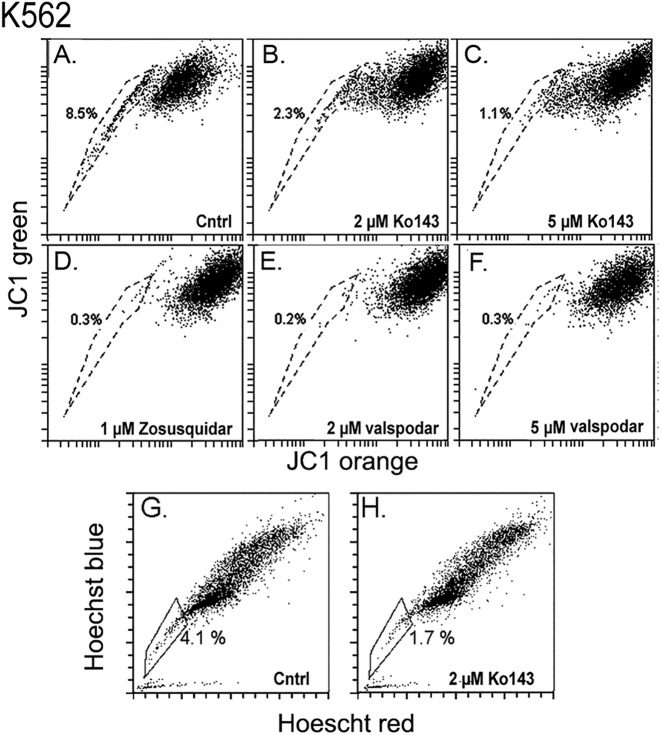
Effect of inhibitors ABC transporters on JC1 or Hoechst exclusion in K562 cells. **A- F.** Cells were incubated for 90 min with JC1 in the absence of an inhibitor (A) or, after pretreatment with 2 μM Ko143 (B), 5 μM Ko143 (C), 1 μM Zosuquidar (D), 2 μM Valspodar (E) or 5 μM valspodar (F). **G and H.** Control cells (G) or cell pretreated with plus 2 μM Ko143 (H) were incubated with Hoechst for 75 min.

Caco-2 and K562 cells also showed robust JC1-SPs (Fig 4 and Fig 5, respectively). In Caco-2 the shRNAs for ABCB1 and ABCG2 caused very similar acceleration of the JC1 uptake (Fig 4A–4C) and, when the JC1 incubation time was extended to reduce the size of the JC1-SP, either Ko143, FTC (Fig 4D–4G) or valspodar (Fig 4H and 4I), each caused essentially complete elimination of the JC1^low^ set. Thus, it would appear that in the Caco-2 cells both ABCB1 and ABCG2 cooperate in the exclusion of JC1 with similar potencies, so that when one of them is abolished, the activity of the other one is not sufficient to generate an effective efflux response. K562 cells also generated a JC1-SP (Fig 5A). The JC1^low^ cohort was greatly reduced by both, 2 or 5 μM Ko143, 1 μM Zosuquidar or 2 or 5 μM valspodar (Fig 5B–5G). However, in these cells, the same 2 μM Ko143 dose that produced near complete inhibition in the Caco-2 cells, caused only a reduction (61 ± 18%, n = 2) in the size of the JC1-SP cohort. Consistent with these results, Caco-2 cells generated a large Hoechst-SP (Fig 5l and 5J) which was fully sensitive to Ko143 (Fig 5K; > 90% reduction in SP, n = 2) but this SP was unaffected by transduction with the ABCB1-A shRNA (Fig 5L). The K562 cells displayed a smaller Hoechst SP than the Caco-2 cells which was only partially affected by Ko143 (Fig 5G and 5H). Finally, when the JC1 stain in either 293T or Caco-2 was chased overnight the degree of JC1 retention as well as the recovery of the full bivariate image following re-incubation with JC1 closely resembled the behavior described in Fig 1 for the HCE cells (not shown).

### Hoechst and DCV effects on JC1 bivariate plot and fluorescent cell images

**Fig 6:**
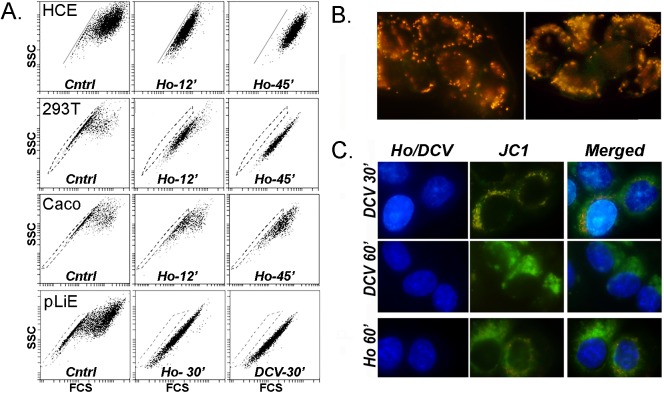
Effect of Hoechst 33342 on JC1 bivariate 525/585 emission plots and fluorescence microscopy in multiple adherent human cells. **A.** Flow cytometry. The four different cell lines, HCE, HEK 293T (293T), Caco-2 (Cacao) and primary limbal epithelial cells were incubated for 60 min with 250 nM JC1 and after dye wash out, were incubated with either 5 ug/ml Hoechst 33342 (Ho) or 5 μM DCV for the indicated times (because of the need to harvest the cell by trypsinization, the times of cell incubation at 37 ⁰ C in the DNA binding dyes are extended by an extra 5–7 min beyond the incubation times stated, before biological activity can be arrested by sample cooling). **B.** Representative fluorescent images of HCE cells incubated for 75 min with JC1 and cultured for 3 h after dye wash up. **C**. Same cells as B but the medium was complemented with 5 μM DCV or 5 μg/ml Hoechst for 30 or 60 min during the last part of the 3 h culture. Right, center and left panels show, respectively, the fluorescence of the DNA dyes alone, JC1 alone and the merged images.

In the next logical step, we attempted to assess the degree of cross- correlation between the JC1-SP and Hoechst-SP cells by sequential incubation of both dyes. Surprisingly, Hoechst or DCV greatly disrupted pre-established bivariate JC1 plots (Fig 6). Hoechst induced changes in the bivariate JC1 emission image in the transformed cell lines HCE, Caco-2 and HEK 293T, and the pcLiE cells resembling those seen after the addition of FCCP in Fig 1. DCV had essentially an identical impact on the JC1 emission pattern to that of Hoechst (Fig 6, pLiE panels Ho-30’ and DCV-30’). The effect of the two DNA intercalating dyes was also examined by fluorescence microscopy in the HCE cells. Time dependent imaging showed that as the Hoechst (blue stain) accumulated in the nuclei (no staining Hoechst staining could be distinguished in mitochondria or elsewhere), the orange fluorescence puncta prevalent in the mitochondria of the control JC1 cells (Fig 6B) gradually reverted to green. By 30 min in DCV, there were only a few orange puncta left (Fig 6C). After 60 min exposure to either DNA intercalating dye, the cytosol presented only green fluorescence.

### JC1-Hoechst SP cross-correlation

**Fig 7:**
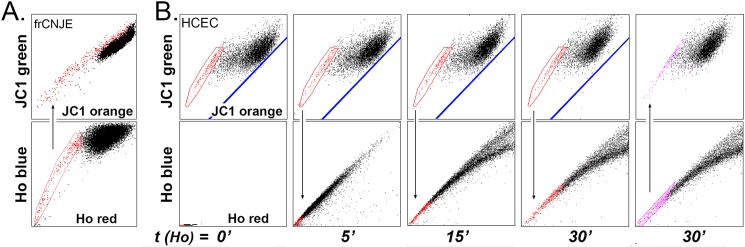
Cross correlation of JC1 and Hoechst exclusion cohorts (SPs) in cells expressing only ABCG2. **A.** Freshly isolated rabbit conjunctiva epithelial cells that have been cultured overnight were simultaneously incubated for 75 min with 250 nM JC1 and 4μg/ml Hoechst. A Hoechst-SP gate was defined in the flow cytometry software under red color coding. In the JC1 image the great majority of these Hoechst-SP cells fall within an identifiable JC1-SP range. **B.** HCE cells incubated for 75 min with JC1 and washed were incubated with 2 μg/ml Hoechst for 5–10 and 30 min, as indicated. The same color coding method described in A was used to track the uptake of Hoechst by the JC1-SP cells. The JC1-SP cells (red) show the slowest accumulation of Hoechst as function of time (arrow down). In the last frame the process of cross correlation was reversed to show that the cohort of cells with the lowest accumulation of Hoechst at 30 min (pink color gate) consists almost exclusively of JC1-SP cells (arrow up). Even with the lowered Hoechst concentration the leftward shift of the main JC1 population (i.e., depolarization) starts developing within 30 min (increased distance from blue line).

Because of the effect of Hoechst and DCV on the JC1 image it was not possible in most cases to assess the degree of cellular overlap (cross correlation) between the Hoechst and JC1 SPs. Only in freshly isolated rabbit conjunctival epithelial cells the JC1 signal degradation by Hoechst was slow or moderate enough to allow simultaneous visualization of both JC1-SP and Hoechst SP. In these cells, the numerically small, but strong Hoechst-SP represents *bona fide* slow cycling stem cells, with clonal ability and radically distinct gene expression profile than the non-SP cells [8, 19]. Simultaneous 75 min co-incubation with 250 nM JC1 and 4 μg/ml Hoechst yielded Hoechst and JC1 SPs amounting to under 1% of the respective populations (Fig 7A). Cross correlation analysis showed that around 92% of the cells in the Hoechst-SP were also contained within the JC1-SP and conversely 88% of the JC1-SP were Hoechst–SP (mean for n = 4). Additionally, to overcome the hindrance to JC1-Hoechst correlation analysis posed by the degradation of the JC1 image by Hoechst in the transformed cells we resorted to a sequential dye incubation protocol. HCE cells were incubated first with JC1 and, following dye wash out, they were incubated with only 2 μg/ml Hoechst, to slow the Hoechst degradation effect on the JC1 image. Cells were then harvested after 5, 10 or 30 min and the JC1-SP and Hoechst SP cross correlation was examined (Fig 7B). Even though, under these limited Hoechst concentration the JC1 image still gradually degraded (see JC1 population shift respect to the defining blue line) and the Hoechst image did not fully developed, it is apparent that the JC1-SP cells has a high degree of cross correlation with the cells that lag in the accumulation of Hoechst (arrows) and hence can be expected to populate the SP when the correct higher concentration and longer incubation are used.

### Effect of JC1 and Hoechst on MMPT, cellular physical parameters, proliferation and cell death

**Fig 8:**
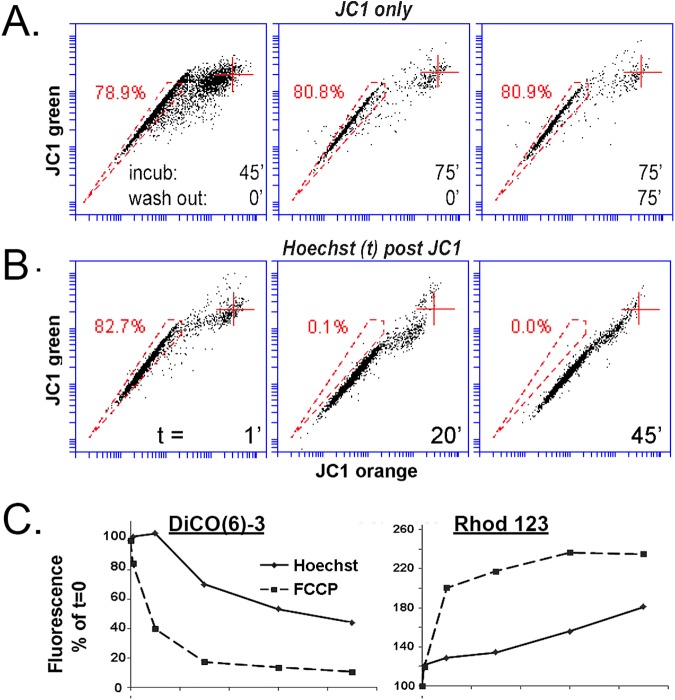
Effect of Hoechst 3342 and FCCP on MMPT as determined by JC1, DiOC_6_-3 or Rho 123 in K562 cells. **A.** A high density K562 cell culture (3 x10^6^ cells/ml) was incubated with 250 nm JC1 for 45 and 75 min as indicated, or incubated for 75 min with JC1, spun down, resuspended in medium for an additional 75 min. **B.** Cells that have been incubated for 75 min with JC1 were complemented with 5μg/ml Hoechst and the changes in the JC1 image were recorded after 1, 20 and 45 min. Note that the SP and nonSP shift in opposite direction along the orange axis. **C.** K562 cell cultures (0.2 x 10^6^ cells/ml) were incubated with 5 nM DiOC_6_-3 for 20 min or 100 nM Rho123 for 60 min. The cultures were then complemented with either 5 μg/ml Hoechst 33342 or 15 μM FCCP and the changes in fluorescence were recorded at time intervals. Both FCCP and Hoechst caused time dependent decreases in DiOC (6)-3 and increases in Rho123 fluorescent emission, respectively. The FCCP effect was more rapid and pronounced. Results are the mean of two experiments.

The effect of the DNA binding dyes on the JC1 image diverged from the effect of FCCP in one visible manner. FCCP solely caused a leftward shift of the main JC1 population to align all the cells on the axis defined by the JC1-SP. This effect is consistent with the interpretation given above for the JC1 image, namely that the rightward shift described in Fig 1 depends on high MMPT, which FCCP abolishes. Hoechst, though, induced this effect initially, but before the full leftward shift could be completed, at later times Hoechst caused a rightward shift that could be interpreted as MMPT hyperpolarization, rather than depolarization (e.g. Fig 6 compares the 12 and 30 min panels for the HCE and 293T cells). Thus, we performed experiments to compare the effect of Hoechst on the JC1 image with its effect on the fluorescent emission of other MMPT sensitive mitochondrial dyes. These experiments were principally carried out in the K562 cells because with suspension cells experiments can be completed using a single cell batch. For these experiments we used a very high cell concentration (Fig 8A). Under these pre-tuned conditions all the available JC1 dye is taken by the cells before the end of the incubation (compare 45 min and 75 min in Fig 8A) and the JC1-SP becomes very large. As shown before for HCE the JC1-SP image remained unchanged during a 75 min wash out period. Hoechst opposite direction shift for the JC1-SP and JC1-nonSP were then easily visualized (Fig 8B). In spite of this puzzling effect, Hoechst (Fig 8C; solid lines) caused changes in the fluorescence of DiOC (6)-3 (increase) and Rho123 (decrease) that paralleled the changes induced by FCCP (dashed lines) and were consistent with MMTP decrease. This result confirms that, as it accumulates in the nuclei Hoechst it nearly simultaneously induces mitochondrial depolarization.

The apparent inhibitory effect of Hoechst on MMPT as reported by the JC1 image change is in line with known induction of toxic reactive oxygen species upon binding of DNA intercalating compounds, including Hoechst [26–28]. For this reason, SP cells isolated using this dye may not be appropriate for the isolation of undamaged live cells. Even if exposed cells survive any short term cytopathic effects of DNA intercalation, the cells may acquire genomic mutations, the chances of which, have been shown to be increased by exposure to UV light during flow cytometry sorting [29, 30]. Thus, JC1 may present an advantage for isolating MDR transporter-rich cells intended for downstream uses, including human organ regeneration. Hence, we considered it important to compare the post incubation effects of the two MDR transport reporting dyes on gross cellular morphological features, cell survival and proliferation.

**Fig 9:**
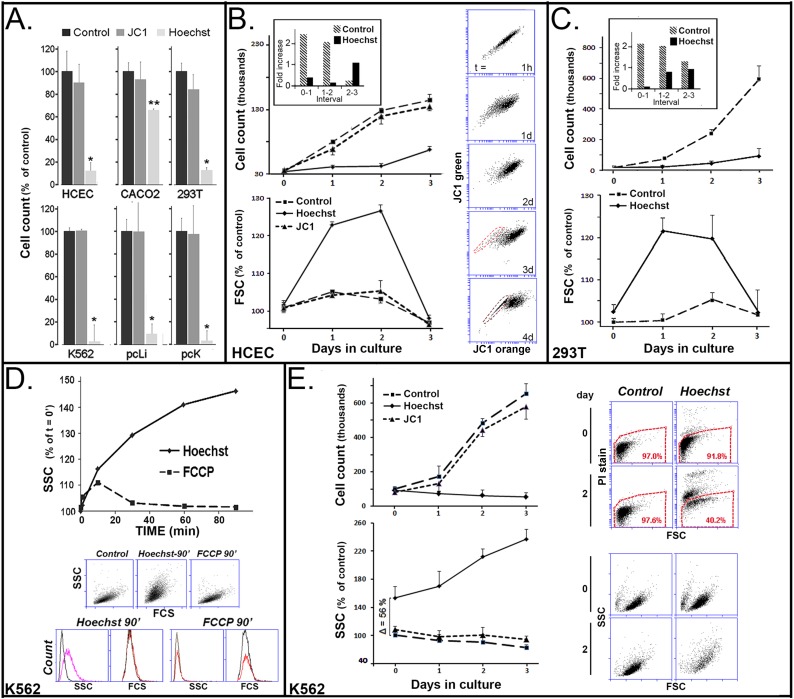
Effect of exposure to JC1 and Hoechst on proliferation of cultured transformed and primary tissue cells. **A.** Adherent cells at a ~ 40% density were treated with 250 nM JC1 or 5 μg/ml Hoechst for 90 min in triplicates or left untreated (Control). Cells were then cultured for 48 h and cells were counted in the Accuri 6. There were no statistical difference between control and JC1-treated cells. * and ** respectively indicate p < 0.01 and p < 0.05 for Hoechst respective to both control and JC1. **B.** HCE cells treated with 5 μg/ml Hoechst, or 250 nM JC1 for 90 min or left untreated. Cells were counted (top line plot), analyzed for relative live cell size (FCS, bottom line plot) and for JC1 accumulation (vertically oriented bivariate JC1 emission plots) every day for 3 to 4 days… The JC1 treatment caused no statistically demonstrable difference in cell count or FCS from control. Both populations increased about two fold for the first and second day (insert). In contrast cell counts for the Hoechst treated cells remain unchanged during these first 2 days. Additionally, FCS underwent a substantial increase and JC1 exclusion activity was abolished during this period. These negative effects waned during days 3 and 4. **C.** 293T cells exposed to Hoechst for 90 min. Note similarity of results with those for HCE cells for both cell count and FCS. **D.** Effect of Hoechst and FCCP on K562 cells. Cells were treated in the same manner used in Fig 5 to examine their effect on MMPT. Values represent the mean of duplicates. The line plot, the flow cytometry FSC/SSC bivariate plots and the SSC and FCS histograms show that Hoechst, but not FCCP, caused a selective increase in SSC during the incubation time without correlated changes in FSC. **E.** Cell count, SSC, fraction of live cells within the cells suspension and SSC/FCS ratios in K562 cells during the post-exposure 3 days. JC1 cell count (top line plot), and SSC values (bottom line plot) for the JC1 exposed cells were indistinguishable from control. In the Hoechst-treated K562 cells, cell count decreased gradually over the three day period while the SSC, which has already increased during the 90 min treatment period (Δ = 56%), continue to increase becoming after 3 days 2.5 higher than the control. This SSC increase occurred without any substantial increase in FCS (SSC/FCS plots). At the same time, the % of dead cells increased every day (PI stain /SSC plots).

Fig 9A displays counts for cells maintained in control conditions vs cells exposed to 250 nM JC1 or 4 μg/ml Hoechst for 90 min two days after dye exposure. In none of the 6 cell types tested were counts for JC1-treated cells statistically different from the control count. In contrast, the exposure to Hoechst was associated with large, statistically significant decreases in the number of cells recovered in all six transformed or tissue-derived cells used in this study. The effect was particularly strong on the K562 cells and the primary keratinocytes. DCV has an effect comparable to that of Hoechst (not shown).

Day by day cell count in HCE and 293T treated cells (Fig 9B and 9C, top line plots) showed that the difference in cell count reflected a large reduction in proliferation rate as result of the nuclear accumulation of Hoechst for between 48 to 72 h. (the decreased in cell count for control and JC1 treated cells between day 2 and 3 shown in the insert frame in Fig 9B, occurred because the cultures reached confluence). In the Hoechst exposed cells, in contrast, cell count was minimally changed during the first 2 days. It started to increase by day 3. At the same time, daily sampling of the culture medium showed that there was no significant release of cells into the medium. Thus, cell death can be ruled out as a factor in the strong reduction of cell count. Consistent with the results of Fig 9A, the count for the HCE cells treated with JC1 was not statistically distinguishable from the count in the control sample through the same 3 days interval (Fig 9B) The same was true for 293T cells (not shown). During this period of growth inhibition the average FSC for both Hoechst treated HCE and 293T cells increased by 30–40% (Fig 9B and 9C, bottom line plots). The SSC/FSC ratio, though, remained constant, indicating that the cells were growing in size consistent with a proliferation arrest. The size increase waned once the cell proliferation re-started by day 3. Intriguingly, during the same period of proliferation arrest, the ABCG2 activity that generates the JC1-SP was curtailed; transport recover fully only by day 4 post exposure (Fig 9B; JC1 bivariate plots on right side).

The very large difference in cell count at 48 h between control and Hoechst exposure in the K562 cells (Fig 9A) hinted at a stronger impact of the DNA dye on these cells. Indeed, unlike the case for HCE and 293T cells, where the cell’s physical parameters were unchanged during the incubation with Hoechst (not shown), in K562 cells, the SSC of live (PI-negative) cells started to increase soon after introduction of the nuclear dye and increased during the whole 90 incubation period without change in FCS (Fig 9D). This selective increase in cell ‘granularity’ is indicative of early stages of cell death, due to crenation of the plasma membrane [31]. FCCP, which has stronger effects on the MMPT than Hoechst (Fig 8), did not cause a similar increase in the SSC (Fig 9D). Thus, the Hoechst effect is either unrelated to MMPT loss or, if related, it involves additional cellular effects that are induced only by Hoechst.

Finally, Hoechst and JC1 treated K562 cells were cultured for the next 3 days as done for the adherent cell lines using single batches from where aliquots were removed every 24 h. During this time the cell counts for the Hoechst treated cells underwent a gradual decreased (Fig 9E; top line plot). The decrease was concurrent with a gradual decrease in the % of live cells within the cell culture suspension (Fig 9; flow cytometry top four plots) and further SCC increases in the surviving cells (Fig 9;, flow cytometry bottom four plots). As for the HCE cells, the post JC1-exposure cell count and SSC/FCS parameters for the K562 cells was not visible different from the control values.

## Discussion

This report examines the application of JC1 for the identification of cells displaying high ABC transporter activity. JC1 exhibits a dual peak emission spectrum and undergoes a bathochromic shift in a concentration dependent manner reflective of monomer aggregation, an effect that is induced by high MMPT. The study shows that the MMPT-driven aggregation occurs only after a certain threshold of dye accumulation and thus, due to its function as a substratum for ATP binding cassette/MDR transporters, incubation of cells carrying these transporters with JC1 yields, under proper incubation conditions, bivariate emission plots incorporating a well-defined subpopulation of cells with high dye extrusion capacity situated to the left (i.e., shorter or ‘bluer’ wavelength) of the main population. Thus, notwithstanding differences in the sites of binding and electronic mechanism for development of bathochromic shifts, this JC1^low^/JC1-SP subpopulation is graphically equivalent to the side population generated by incubation of cells with the DNA intercalating dyes, Hoechst and DCV.

JC1 usage presents a number of biological and practical advantages to the reliance on the DNA binding dyes. Firstly, JC1 binds exclusively to mitochondria on non DNA sites, thereby avoiding genotoxic risks. Secondly, the binding is surprisingly strong; it takes almost a full day to reduce average emission intensity to half. Thirdly, in spite of this tight attachment, JC1 does not affect the MMPT, the critical parameter for the generation of ATP. Fourthly, the absence of effect on MMPT, FSC/SSC and rate of proliferation indicates the absence of any substantial toxic effect for JC1 in the conditions need to identify SPs. Fifthly, JC1 measurements require only the basic argon laser; application of Hoechst and DVC require either UV or violet lasers that are frequently not available in developing countries or smaller academic facilities.

In terms of the biological usefulness, JC1 apparent disadvantage is its lack of specificity between ABCG2/BRCP and ABCB1/p-glycoprotein. For the identification of ABCG2-rich somatic stem cells the high specificity of Hoechst for ABCG2 is a clear advantage. DCV has been shown to be a substratum for both ABCG2 and ABCB1 [32, 33]. Nevertheless, as shown in Fig 7, in cells with little or no ABCB1 activity, JC1 and Hoechst generate SPs with a reciprocal overlap of about 90%. The availability of ABCB1 and ABCG2 selective inhibitors should help surmount the selectivity hindrance to the application of JC1 on the isolation of cancer cells over expressing one or the other transporter and somatic stem cells overexpressing ABCG2. Finally, in our studies with HCE cells the JC1-SP (Fig 2A; 8.7%) was shown to be larger than the Hoechst-SP (Fig 2F; 2.3%). On the surface this is a confounding result. Yet, as shown in Fig 1G the size of the SPs is a function of JC1 concentration and time of incubation, hence equal magnitudes can be achieved by manipulating these parameters with JC1. In fact the same dependence on the SP size on time and concentration of incubation hold true for Hoechst too (unpublished)

Additionally, our studies revealed heretofore unidentified rapid acute effects of Hoechst on cell function. Along with the accumulation in the nucleus, Hoechst induced a decay of the MMPT similar to that induced by the mitochondrial uncoupler FCCP, for the first 48 to 72 h post dye exposure caused major delays in cell cycling, increased cell size and inhibition of ABCG2 efflux activity in multiple cell lines. Furthermore, in at least one cell line (K562), Hoechst induced an immediate change in SSC which usually heralds initial stages of cell death and actually cell death over the next 3 days. The fact that the immediate Hoechst effect on SSC appears to be unrelated to the MMPT diminution, that Hoechst causes complex biphasic changes in the JC1 image within its accumulation period and that during the slowed proliferation period there appear to be no ABCG2 transport in the HCE cells, are three observations that suggest complex deleterious impact of Hoechst on cells beyond the reduction of MMPT. Intriguingly, Hoechst has been reported to have minimal effect on the colony formation efficiency of certain freshly isolated hematopoietic precursors [34]. This resiliency may be related to the slow cycling or relative quiescence of both hematopoietic and ocular surface epithelial precursors; cytopathic effects may not occur because these cells become activated in culture only after Hoechst impact wanes. Nevertheless, given the observed effects in cultured cells comparing clonogenic capacity of primary cells isolated with either Hoechst or JC1 to see whether, in fact, Hoechst has no partial detrimental effects seem warranted.

In summary, the use of JC1 for the isolation of high transport activity cells obviates the disadvantages associated with the use of DNA binding dyes including the need of expensive cytometry instrumentation and potential toxicity directly related to DNA intercalation and the need to use UV excitation. Adult stem and progenitor cells or cancer stem cells incorporating either of the two MDR transporters can be isolated by flow cytometry without significant impact in their physiological or biological condition. Actual verification that JC1^low^ cells has *in vivo* regenerative capacity in a test such as hematopoietic bone marrow repopulating capacity [35] is pending.
